# A Gradient-Penalized Conditional TimeGAN Combined with Multi-Scale Importance-Aware Network for Fault Diagnosis Under Imbalanced Data

**DOI:** 10.3390/s25226825

**Published:** 2025-11-07

**Authors:** Ranyang Deng, Dongning Chen, Chengyu Yao, Dongbo Hu, Qinggui Xian, Sheng Zhang

**Affiliations:** 1 School of Mechanical Engineering, Yanshan University, Qinhuangdao 066004, China; rydeng@stumail.ysu.edu.cn (R.D.); 18629448551@163.com (Q.X.);; 2 Hebei Provincial Key Laboratory of Heavy Machinery Fluid Power Transmission and Control, Yanshan University, Qinhuangdao 066004, China; 3 Hebei Key Laboratory of Industrial Computer Control Engineering, Yanshan University, Qinhuangdao 066004, China; chyyao@ysu.edu.cn; 4 Citic Heavy Industries Co., Ltd., Luoyang 471000, China

**Keywords:** rotating machinery, fault diagnosis, data augmentation, time-series generative adversarial network

## Abstract

In real-world industrial settings, obtaining class-balanced fault data is often difficult. Imbalanced data across categories can degrade diagnostic accuracy. Time-series Generative Adversarial Network (TimeGAN) is an effective tool for addressing one-dimensional data imbalance; however, when dealing with multiple fault categories, it faces issues such as unstable training processes and uncontrollable generation states. To address this issue, from the perspective of data augmentation and classification, a gradient-penalized Conditional Time-series Generative Adversarial Network with a Multi-Scale Importance-aware Network (CTGAN-MSIN) is proposed in this paper. Firstly, a gradient-penalized Conditional Time-Series Generative Adversarial Network (CTGAN) is designed to alleviate data imbalance by controllably generating high-quality fault samples. Secondly, a Multi-scale Importance-aware Network (MSIN) is constructed for fault classification. The MSIN consists of the Multi-scale Depthwise Separable Residual (MDSR) and Scale Enhanced Local Attention (SELA): the MDSR network can efficiently extract multi-scale features, while the SELA network is capable of screening out the most discriminative scale features from them. Finally, the proposed method is validated using the HUST bearing dataset and the axial piston pump dataset. The results show that under the data imbalance ratio of 15:1, the CTGAN-MSIN achieves diagnostic accuracies of 98.75% and 96.50%, respectively, on the two datasets and outperforms the comparison methods under different imbalance ratios.

## 1. Introduction

As a core element of sophisticated industrial systems, the sustained and reliable functioning of rotating machinery is vital for maintaining both operational safety and productivity [1,2]. Typical rotating machinery, such as bearings and piston pumps, is often subjected to various pressures and loads, making it susceptible to failures like wear and fatigue, which may lead to shutdowns or even safety accidents [3,4]. In real-world industrial scenarios, rotating machinery is characterized by short operational durations under fault conditions and difficulties in obtaining fault samples, resulting in a scarcity of fault data. An imbalance in the data arises from the disproportionate representation of normal samples compared to fault samples [5]. Under data imbalance conditions, diagnostic models are often biased toward the majority class, overlooking the rare one and thus diminishing diagnosis accuracy [6]. Therefore, investigating rotating-machinery fault diagnosis amid data imbalance is of pronounced practical value.

The application of data augmentation techniques can counteract data imbalance problems, leading to improved performance in fault diagnosis systems [7]. Common data augmentation techniques mainly fall into two categories: methods based on data sampling and those relying on data generation [8]. The data sampling-based methods include oversampling [9], undersampling [10], and interpolation-based techniques [11]. By undersampling the majority classes and oversampling the minority classes, the class distribution can be balanced. However, such methods suffer from limitations such as limited representational capacity and insufficient data diversity. Data generation-based methods include approaches such as Variational Autoencoder (VAE) [12] and Generative Adversarial Network (GAN) [13]. However, data generated by VAE often exhibits limited diversity and authenticity, along with the vanishing gradient problem in long sequences [14]. GAN employs an adversarial training mechanism where the generator and discriminator undergo dynamic game-theoretic optimization. This framework effectively captures the underlying data distribution characteristics, enabling the generation of synthetic data that maintains high statistical consistency with real data. GAN has now become the primary method for data augmentation.

Lately, a series of enhanced GAN-based techniques has continually surfaced, such as Auxiliary Classifier GAN (ACGAN) [15], Deep Convolutional GAN (DCGAN) [16], Conditional GAN (CGAN) [17], Wasserstein GAN (WGAN) [18], and so on. Additionally, some scholars have explored innovative approaches that combine GANs with other network architectures. For example, Chen et al. [19] proposed integrating a VAE with an attention mechanism as the generator of the GAN, thereby enhancing its attention on fault-relevant features. Li et al. [20] proposed an augmentation strategy combining an adaptive diffusion model with GAN, which enhances the diversity of fault samples. Yoon et al. [21] proposed Time-series Generative Adversarial Network (TimeGAN), a novel generative framework specifically designed for synthesizing realistic one-dimensional time-series data, as opposed to generating images directly. This model synergistically combines the strengths of GAN with supervised learning, enabling the generation of high-fidelity temporal data while preserving the original time-dependent structural characteristics. Shi et al. [22] proposed an augmentation strategy for imbalanced datasets based on a Wasserstein Temporal Generative Adversarial Network with Gradient Penalty (WTGAN-GP), and validated its performance in addressing class imbalance using real-world air compressor fault data from industrial plants. Sim et al. [23] employed TimeGAN to address data imbalance issues in real-world industrial datasets and developed a CNN-LSTM hybrid network, successfully forecasting equipment that remains useful across diverse load scenarios. However, despite demonstrating advantages in time-series data generation, TimeGAN still has certain limitations: (1) Due to the dynamic adversarial training mechanism, it is prone to mode collapse or convergence difficulties. (2) As a general time-series generation framework, the TimeGAN method still faces critical challenges such as training instability and uncontrollable generation outcomes.

The design of feature extraction and classification networks is a critical component in fault diagnosis. Currently, most imbalanced fault diagnosis studies primarily focus on data augmentation techniques, while subsequent fault diagnosis tasks often rely solely on some basic benchmark methods. For instance, Ye et al. [24] developed a feature fusion deep convolutional generative adversarial network and subsequently utilized convolutional stacks and fully connected layers to achieve fault classification in aircraft engines. Wang [17] and Chen et al. [19] employed variants of GAN-based networks for data augmentation and then implemented fault classification using deep convolutional neural networks and Softmax. Deep Learning (DL) algorithms have gained broad traction in recent years for their capabilities in automatic feature extraction, multi-level feature learning, and strong generalization performance [25]. Common DL approaches include Convolutional Neural Network (CNN) [26], Recurrent Neural Network (RNN) [27], and Transformer Network [28]. Compared with CNN, RNN and Transformer models exhibit higher architectural complexity and require substantially more time for network training. Due to the inherent multi-timescale characteristics in rotating machinery signals, the Multi-scale Convolutional Neural Network (MSCNN) has been widely adopted for analysis [29]. As the number of network layers increases and the scale of parameters expands, MSCNN-based models tend to suffer from gradient vanishing or explosion due to the excessive number of nonlinear layers. When gradient vanishing occurs, parameter updates essentially cease, and even in cases where extreme gradient problems are avoided, parameter optimization becomes progressively more difficult with increasing network depth [30]. Moreover, deeper networks inevitably bring greater complexity and longer training times. Therefore, MSCNN still faces some challenges in practical applications.

Residual Network (ResNet) offers an effective remedy for gradient vanishing and explosion in deep neural networks. Liu et al. [31] proposed a multiscale kernel-based residual CNN, incorporating residual learning modules with multiscale kernels to accomplish motor fault diagnosis. Xu et al. [32] developed a residual network architecture integrated with a multi-scale attention mechanism for diagnosing faults in variable displacement pumps. However, existing multi-scale feature extraction methods focus on expanding the broader receptive field, while neglecting the varying differences in features across different scales.

To address the aforementioned challenges, a gradient-penalized Conditional Time-series Generative Adversarial Network (CTGAN) with a Multi-scale Importance-aware Network (MSIN) is proposed in this paper, collectively named CTGAN-MSIN. This paper’s principal contributions are summarized below:

(1) A time-series data augmentation method named the CTGAN network is proposed in this paper. It introduces Wasserstein distance with gradient penalty as the loss function, effectively mitigating the mode collapse issue commonly encountered in traditional TimeGAN network training. By injecting category labels into both the generator and discriminator, this approach effectively overcomes the limitations of uncontrollable outputs in original TimeGAN. It enables the controlled generation of multi-source fault samples, providing a precise data augmentation solution for fault diagnosis.
(2) The MDSR block is proposed to efficiently capture multi-scale feature representations. This module employs multiple branches of depthwise separable convolutions, which significantly reduce computational complexity while accurately capturing multi-scale features. Additionally, MDSR incorporates a residual connection mechanism that effectively preserves the integrity of original features while mitigating gradient vanishing.
(3) To address the issue of low diagnostic accuracy caused by differences in the importance of features across different scales, a Scale Enhanced Local Attention (SELA) module is proposed. The SELA module enhances the expressive capability of features across positional and scale dimensions, enabling the model to precisely focus on the most discriminative scale features. This has significantly improved both recognition accuracy and robustness.
(4) This paper proposes the CTGAN-MSIN network from the perspectives of data augmentation and classification. CTGAN resolves the issues of unstable training and uncontrollable generation outcomes in TimeGAN, effectively accomplishing data augmentation tasks. The MSIN integrates both MDSR and SELA modules to extract comprehensive and highly discriminative features. This model effectively addresses the issue of low diagnostic accuracy in classical MSCNN, which arises from its neglect of the important differences among features at different scales. Practicality and effectiveness of the CTGAN-MSIN are validated using both the public bearing dataset and the self-collected axial piston pump dataset.

The structure of this paper is outlined below: Section 2 offers a concise overview of related theories; Section 3 elaborates on the CTGAN-MSIN methodology, while Section 4 verifies its superiority through two case studies; and Section 5 summarizes the conclusions.

## 2. Preliminaries

### 2.1. Time-Series Generative Adversarial Network

**Figure 1:**
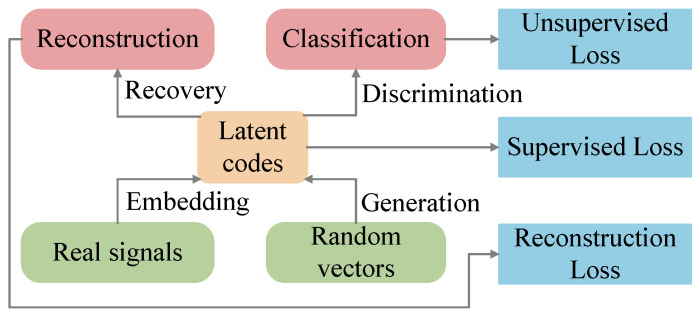
The structure of TimeGAN.

The Time-series Generative Adversarial Network (TimeGAN) is a temporal generative adversarial network proposed by Yoon et al. in 2019 [21]. By integrating adversarial training with supervised learning, TimeGAN effectively generates high-fidelity synthetic data that preserves temporal dependency structures, thus addressing the limitations of traditional GANs in time-series data modeling. As illustrated in Figure 1, the TimeGAN is built upon four essential modules: an embedding, a recovery, a sequence generator, and a sequence discriminator. The core of TimeGAN lies in the collaborative training that combines an auto-encoding component (the embedding module and the reconstruction module) with an adversarial component (sequence generation and sequence discrimination).

Static characteristics *S* and temporal characteristics *Ω* are two characteristics of time-series data. *s* and *ω* are random variables belonging to *S* and *Ω*, respectively. Embedding and recovery networks mediate the feature-latent space transformation, allowing an adversarial network of temporal dynamics in condensed representations. Denote the feature space as *H_S_* and its associated latent space as *H_Ω_*. Define the embedding network as *e*: $S\times \prod_{t}\Omega \to H_{S}\times \prod_{t}H_{\Omega }$. The *e* module transforms both static and temporal features into latent codes *h_S_* and *h_t_*, respectively. Implement the embedding function *e* with a recurrent neural network (RNN).

(1) $$h_{S}=e_{S}\left(s\right),h_{t}=e_{\Omega }\left(h_{S},h_{t-1},\omega_{t}\right)$$

where *e_S_* and *e_Ω_* represent the embedding network for static and temporal features, respectively. 

The recovery network is denoted as *r*, which corresponds to the embedding network. The expression for *r* is $H_{S}\times \prod_{t}H_{\Omega }\to S\times \prod_{t}\Omega$. The *r* decodes latent codes back into static features $\tilde{s}$ and temporal features ${\tilde{\omega }}_{1:\;T}=r(h_{S},h_{1:\;T})$. A feedforward neural network is employed to realize the recovery function *r*.

(2) $$\tilde{s}=r_{S}\left(h_{S}\right),{\tilde{\omega }}_{1:T}=r_{\Omega }\left(h_{t}\right)$$

where *r_S_*: *H_S_*→*S* and *r_Ω_*: *H_Ω_*→*Ω* correspond to functions that project latent representations back to original feature spaces, with *r_S_* for static embeddings and *r_Ω_* for temporal embeddings.

Rather than the direct generation of sequences, the generator of TimeGAN first operates within the embedded latent space. Vectors are randomly sampled from known domains *Z_S_* and *Z_Ω_* as inputs to generate *H_S_* and *H_Ω_*, with the generation process defined by function *g*: $Z_{S}\times \prod_{t}Z_{\Omega }\to H_{S}\times \prod_{t}H_{\Omega }$. Latent codes ${\hat{h}}_{S},{\hat{h}}_{1:T}=g\left(z_{s},z_{1:T}\right)$ are generated by transforming static and temporal random vectors through the generation function *g*. The generation function *g* is implemented with the RNN

(3) $${\hat{h}}_{S}=g_{S}\left(z_{s}\right),{\hat{h}}_{t}=g_{\Omega }\left({\hat{h}}_{S},{\hat{h}}_{t-1},z_{t}\right)$$

where *g_S_*: *Z_S_*→*H_S_* and *g_Ω_*: *H_S_*×*H_Ω_*×*Z_Ω_*→*H_Ω_* denote the generator networks for static and temporal features, respectively. The generation of *z_s_* allows for sampling from an arbitrary distribution, while *z_t_* is governed by a stochastic process.

Define the discriminator function as *d*: $H_{S}\times \prod_{t}H_{\Omega }\to \left[0,1\right]\times \prod_{t}\left[0,1\right]$. The discriminator returns the classifier ${\tilde{y}}_{S},{\tilde{y}}_{1:T}=d\left(h_{S},h_{1:T}\right)$ after receiving both static and temporal latent codes. A bidirectional RNN capped by a feedforward output layer serves as the discriminator *d*.

(4) $${\tilde{y}}_{S}=d_{S}\left({\tilde{h}}_{S}\right),{\tilde{y}}_{t}=d_{\Omega }\left({\overset{\leftarrow }{u}}_{t},{\vec{u}}_{t}\right)$$

where ${\vec{u}}_{t}={\vec{c}}_{\Omega }\left({\tilde{h}}_{S},{\tilde{h}}_{t},{\vec{u}}_{t-1}\right)$ denotes the forward hidden state sequence, whereas ${\overset{\leftarrow }{u}}_{t}={\overset{\leftarrow }{c}}_{\Omega }\left({\tilde{h}}_{S},{\tilde{h}}_{t},{\overset{\leftarrow }{u}}_{t+1}\right)$ denotes the backward counterpart; ${\vec{c}}_{\Omega }$ and ${\overset{\leftarrow }{c}}_{\Omega }$ are the hidden states from the preceding and succeeding time steps, respectively; and *d_S_* and *d_Ω_* serve as the output layer classification functions for static and temporal features.

During training, the TimeGAN framework employs three separate loss functions for network optimization: reconstruction loss (*L_R_*), unsupervised loss (*L_U_*), and supervised loss (*L_S_*). These loss functions are formulated mathematically below

(5) $$\begin{array}{l} L_{R}=E_{s,\omega_{1:\;T\sim P}}\left[{\left\|s-\tilde{s}\right\|}_{2}+\sum_{t}{\left\|\omega_{t}-{\tilde{\omega }}_{t}\right\|}_{2}\right]\\ L_{U}=E_{s,\omega_{1:\;T\sim P}}\left[logy_{S}+\sum_{t}logy_{t}\right]+E_{s,\omega_{1:T\sim \tilde{P}}}\left[log(1-{\hat{y}}_{S})+\sum_{t}log(1-{\hat{y}}_{t})\right]\\ L_{S}=E_{s,\omega_{1:\;T\sim P}}\left[{\sum_{t}\left\|h_{t}-g_{\Omega }(h_{S},h_{t-1},z_{t})\right\|}_{2}\right] \end{array}$$

### 2.2. Depthwise Separable Convolutional Network

**Figure 2:**
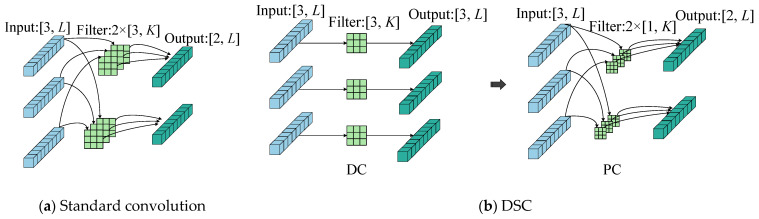
Structural diagrams of standard convolution and DSC.

The Depthwise Separable Convolution (DSC) was proposed by Chollet in 2017, and its structure is a combination of Depthwise Convolution (DC) and Pointwise Convolution (PC) [33]. Compared to standard convolution, it significantly improves the computational efficiency of the model [34]. DC performs independent convolutions on each input channel, generating output feature maps with a quantity consistent with that of the input channels. PC employs 1 × 1 convolution kernels for both cross-channel feature fusion and flexible output channel dimension adjustment. Figure 2 illustrates the architectural comparison between standard convolution and DSC (DC and PC). Unlike standard convolution, which simultaneously processes all input channels, DSC significantly reduces computational complexity. The parameter counts for standard convolution and DSC are calculated as follows

*P*_Conv_ = *C* × *M* × *K* (6)

*P*_DSConv_ = *C* × *K* + *C* × *M* (7)

where *P*_Conv_ represents the standard convolution, and *P*_DSConv_ represents the DSC. Let *C*, *M*, and *K* represent the input channel count, output channel count, and kernel size, respectively.

### 2.3. Efficient Local Attention Module

The attention mechanism is a technique that mimics human cognitive processes, enabling the model to dynamically learn the weights of each input region and highlight only the most salient features. The Efficient Local Attention (ELA) module can accurately focus on key regions, significantly enhancing feature representation capabilities while maintaining its lightweight characteristics [35].

The calculation process for ELA is as follows:

For input *X* of dimensions *C* × *H* × *W*, horizontal and vertical averaging pooling is performed on each channel using pooling kernels of dimensions (*H*, 1) and (1, *W*), respectively. Here, *C* represents the number of channels, while *H* and *W* denote the height and width of the image. The pooling calculations for the *c*-th channel at height *h* and width *w* are given by Equations (8) and (9), respectively.

(8) $$z_{c}^{h}(h)=\frac{1}{W}\sum_{0\leq i<W}x_{c}(h,i)$$

(9) $$z_{c}^{w}(w)=\frac{1}{H}\sum_{0\leq j<H}x_{c}(j,w)$$

where $z_{c}^{h}(h)$ and $z_{c}^{w}(w)$ represent the sequence signal outputs at height *h* and width *w* of the *c*-th channel, respectively, so one-dimensional convolution is used to capture positional details along both horizontal and vertical directions.

To enhance the model’s generalization capability, the Batch Normalization (BN) layer is replaced with a Group Normalization (GN) layer, yielding attention representations in the horizontal and vertical directions

(10) $$y^{h}=\sigma (G_{n}(F_{h}(z^{h})))$$

(11) $$y^{w}=\sigma (G_{n}(F_{w}(z^{w})))$$

where *σ* represents a nonlinear function, *G_n_* represents the GN layer, and *F_h_* and *F_w_* denote the horizontal and vertical one-dimensional convolutions, respectively. *y^h^* and *y^w^* represent horizontal and vertical position attention, respectively. The final output *Y* of the ELA module is obtained by computing *y^h^* and *y^w^* through the Softmax function, expressed as

*Y* = *x*_*c*_ × *y*^*h*^ × *y*^*w*^ (12)

## 3. Proposed Method

### 3.1. Gradient-Penalized Conditional Time-Series Generative Adversarial Network

TimeGAN employs the Jensen–Shannon (JS) divergence as the metric to quantify the dissimilarity between real and generated sequences. To measure the discrepancy between real and generated data, when the two distributions have no overlap, the JS divergence converges to the constant log 2, causing gradient vanishing during training [22]. Meanwhile, TimeGAN suffers from the issue of uncontrollable generated samples. To address these issues, this paper proposes an improved model termed the gradient-penalized Conditional TimeGAN (CTGAN).

Firstly, in the CTGAN, the Wasserstein distance is introduced as a new metric. Its advantage lies in providing stable gradient information, even under substantial distributional divergence between generated and real data, thereby effectively mitigating the vanishing gradient problem. Additionally, a gradient penalty mechanism is replaced by weight clipping, preventing drastic weight fluctuations and thereby reducing issues of vanishing or exploding gradients. This results in a smoother training process. Therefore, the unsupervised loss function ${\hat{L}}_{U}$ of CTGAN is

(13) $${\hat{L}}_{U}=-E_{s,\omega_{1:T\sim P}}\left[y_{S}+\sum_{t}y_{t}\right]+E_{s,\omega_{1:T\sim \tilde{P}}}\left[y_{S}+\sum_{t}y_{t}\right]+\lambda E_{\tilde{s},{\tilde{\omega }}_{1:T}}{\left[{\left\|\nabla_{\tilde{s},{\tilde{\omega }}_{1:T}}({\hat{y}}_{s}+\sum_{t}{\hat{y}}_{t})\right\|}_{2}-1\right]}^{2}$$

(14) $$\tilde{s},{\tilde{\omega }}_{1:T}=\varepsilon (s+\omega_{t})+(1-\varepsilon )(y_{s}+\sum_{t}y_{t})∼{\tilde{p}}_{s,\omega_{1:T}}$$

where *ε* denotes a randomly generated value within the interval (0,1), *λ* represents the weight of the gradient penalty, and ${\left[{\left\|\nabla_{\tilde{s},{\tilde{\omega }}_{1:T}}({\hat{y}}_{s}+\sum_{t}{\hat{y}}_{t})\right\|}_{2}-1\right]}^{2}$ represents the gradient penalty term.

Secondly, the class-conditional labeling mechanism is introduced into CTGAN to generate class-specific fault samples. Then the embedding network is improved to

(15) $$h_{S}=e_{S}\left(s,class\_label\right),h_{t}=e_{\Omega }\left(h_{S},h_{t-1},\omega_{t},class\_label\right)$$

The generator and discriminator are improved through the following modifications

(16) $${\hat{h}}_{S}=g_{S}\left(z_{s},class\_label\right),{\hat{h}}_{t}=g_{\Omega }\left({\hat{h}}_{S},{\hat{h}}_{t-1},z_{t},class\_label\right)$$

(17) $${\tilde{y}}_{S}=d_{S}\left({\tilde{h}}_{S},class\_label\right),{\tilde{y}}_{t}=d_{\Omega }\left({\overset{\leftarrow }{u}}_{t},{\vec{u}}_{t},class\_label\right)$$

The supervision loss function is improved to

(18) $${\hat{L}}_{S}=E_{s,\omega_{1:T\sim P}}\left[{\sum_{t}\left\|h_{t}-g_{\Omega }(h_{S},h_{t-1},z_{t},class\_label)\right\|}_{2}\right]$$

### 3.2. Multi-Scale Importance-Aware Network

In scenarios with imbalanced data, the design of feature extraction and classification networks is a critical step in fault diagnosis. Multi-scale CNN (MSCNN) has emerged as a predominant approach in this field due to its unique capability to concurrently extract multi-temporal-scale features, which aligns perfectly with the multi-scale characteristics of rotating machinery signals. However, features at different scales have varying degrees of importance. Concatenating and fusing features from different scales may result in the loss of critical scale-specific feature information and consequently reduce diagnostic accuracy.

To tackle this challenge, this study innovatively proposes a Multi-scale Importance-aware Network (MSIN) framework for fault classification. MSIN employs a multilevel feature extraction mechanism, which can effectively capture fault feature representations at different scales. Furthermore, a Scale Enhanced Local Attention (SELA) module is introduced that utilizes an adaptive weight allocation mechanism, enabling the model to direct its attention with high precision to the most discriminative scale features. This design not only achieves dynamic assessment of the importance of features at different scales but also significantly enhances feature amplification and classification performance.

(a) Multi-scale depthwise separable residual block

**Figure 3:**
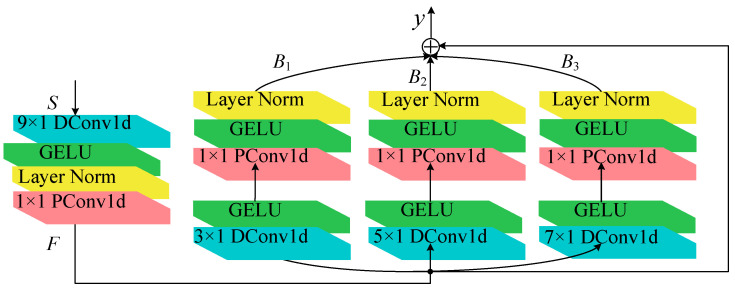
Structural diagram of MDSR.

Drawing inspiration from the Inception module [36] and residual design of ResNet, the Multi-scale Depthwise Separable Residual (MDSR) module is proposed. By leveraging DSC and residual connection techniques, the MDSR effectively mitigates the issues of gradient vanishing and explosion while efficiently guiding the classification model to extract rich fault features. A schematic diagram of the MDSR is presented in Figure 3.

The input signal *S* to the MDSR module is a one-dimensional signal with dimensions (*C*, *L*), where *C* and *L* represent the number of channels and the signal length, respectively. The MDSR operation process is as follows.

Firstly, a 9 × 1 large-kernel DSC module is designed to initially capture broader-scale fault features, whose mathematical expression is

(19) F=σ(LN(DSConv1d(S)))

where *F* denotes the obtained feature. To accelerate network convergence and mitigate overfitting, Layer Normalization (LN) is adopted. The activation function utilized is the smoother GELU.

Subsequently, we constructed a multi-branch parallel structure comprising three independent processing branches. Each branch follows the following processing workflow: first, different-sized DC is applied (with kernel sizes of 3 × 1, 5 × 1, and 7 × 1 for each branch), followed by LN and GELU activation function processing.

Then, PC (1 × 1 convolutions) is performed, and the GELU activation function is applied again. The expression for this process is

(20) $$B_{i}=\sigma (PConv1d(\sigma (LN(DConv1d(F)))))$$

where *B_i_* represents the characteristics of different branches, *i* = 1, 2, 3. The shape of *B_i_* is (*C*/*r*, *L*), where *r* is the reduction ratio. Channel parameters can be reduced by decreasing *r*.

Finally, the output features of the three branches are concatenated along the scale to form a two-dimensional multi-scale feature map *I_m_*∈*R^C^*^/*r*×*L*×*B*^, where *B* denotes the number of multi-scale kernels. To preserve critical information from the original input and mitigate gradient vanishing in deep architectures, residual connections are incorporated. This operation concatenates features *F* and *I_m_* to produce the output feature *y*. The expressions for the multi-scale feature map *I_m_* and the final output feature *y* are given by Equations (21) and (22), respectively,

*I*_m_ = [*B*_1_, *B*_2_, *B*_3_] (21)

*y* = [*I*_m_, *F*] (22)

In the above equation, *y*∈*R^C^*^/*r*×*N*×*L*^, where *N* = *B* + 1.

(b) Scale enhanced local attention module

**Figure 4:**
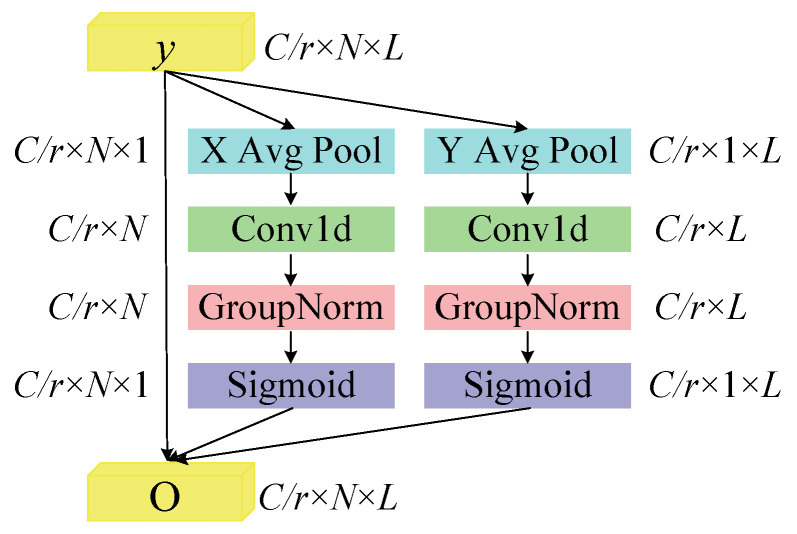
The proposed SELA module.

Considering that features at different scales have varying degrees of importance, a Scale Enhanced Local Attention (SELA) module is proposed. This module draws inspiration from ELA for generating precise positional attention maps through average pooling along the height and width directions. For the multi-scale features *y* generated by MDSR, it performs average pooling along both the length and scale dimensions, thereby generating attention maps containing distinct scale and positional information. The SELA module effectively enhances the expressive power of features across different positions and scales, precisely guiding the model to focus on the most discriminative scale features. Figure 4 illustrates the specific operational flow of the SELA module and the corresponding dimensional changes.

Input the one-dimensional vibration signal into the MDSR module to obtain the multiscale feature *y* as input for SELA. Perform average pooling on each channel using pooling kernels of sizes (*N*, 1) and (1, *L*). The pooling computation process for the *c*-th channel at scale *n* is as follows

(23) $$z_{c}^{n}(n)=\frac{1}{L}\sum_{0\leq i<L}y_{c}(n,i)$$

The pooling calculation process for the *c*-th channel at length *l* is as follows

(24) $$z_{c}^{l}(l)=\frac{1}{N}\sum_{0\leq j<N}y_{c}(j,l)$$

One-dimensional convolutions are employed to enhance scale and positional information, followed by GN to augment the feature information. The resulting horizontal attention *g^b^* and vertical attention *g^l^* expressions are as follows

(25) $$g^{b}=\sigma (G_{n}(F_{b}(z^{b})))$$

(26) $$g^{l}=\sigma (G_{n}(F_{l}(z^{l})))$$

In the above description, *F_b_* and *F_l_* denote one-dimensional convolution operations. Applying Equation (27) yields the SELA output *O*

*O* = *y*_*c*_ × *g*^*b*^ × *g*^*l*^ (27)

(c) Multi-scale importance-aware network

**Figure 5:**
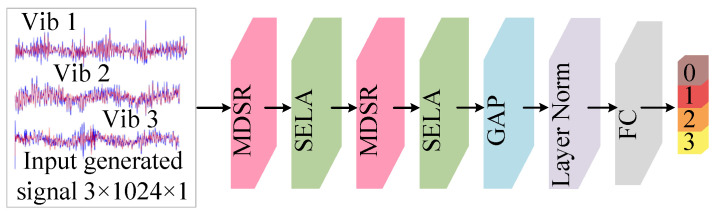
Framework diagram of the MSIN.

**Figure 6:**
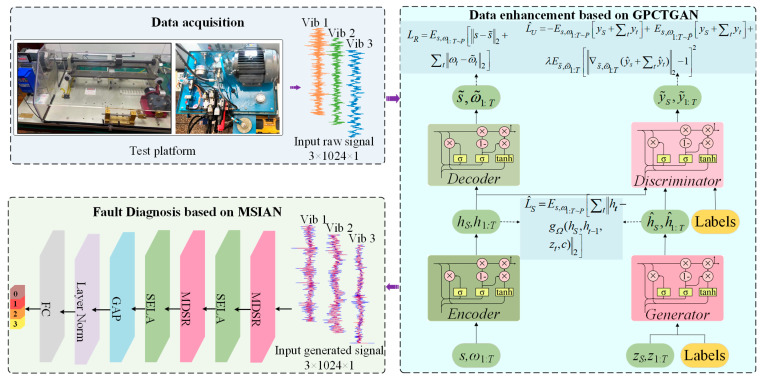
The process of CTGAN-MSIN.

A novel architecture, the Multi-scale Importance-aware Network (MSIN), is proposed in this subsection. The framework of MSIN is illustrated in Figure 5, and its diagnostic framework consists of the MDSR module and the SELA module. The MDSR efficiently extracts features at different scales, while the SELA module performs adaptive weighting on the multi-scale features extracted by the MDSR to emphasize discriminative key scale features. The classifier compresses feature dimensions using Global Average Pooling (GAP), then maps them through a Fully Connected (FC) layer, and finally uses the Softmax function to output the probability distribution of fault categories. The entire process of CTGAN-MSIN is illustrated in Figure 6.

## 4. Case Studies

Two cases of rotating machinery are used to demonstrate the data augmentation and fault diagnosis capabilities of the CTGAN-MSIN, namely the HUST Bearing public dataset [37] and the laboratory axial piston pump self-collected dataset.

### 4.1. Case 1: HUST Bearing Dataset

#### 4.1.1. Description of HUST Bearing Test

The HUST bearing failure test rig integrates a speed control, a motor, a shaft, and three acceleration sensors. The test collected vibration signals in three directions, with four different speed conditions set: 3900 rpm, 4200 rpm, 4500 rpm, and 4800 rpm. Data were acquired at a sampling frequency of 25.6 kHz for a duration of 10.2 s. Table 1 presents the specific fault types of the bearing and the corresponding data-splitting details adopted in this work. To simulate data imbalance scenarios, we constructed 150 samples for the normal state and 10 samples for each fault state. Each sample contains at least two rotation cycles, thus comprising 1024 data points.

#### 4.1.2. Data Generation and Evaluation in the HUST Bearing Dataset

This study utilizes the proposed CTGAN model to augment fault state data. In the CTGAN architecture, GRU is employed as the recurrent neural network layer. Dropout layers are incorporated into the network to mitigate overfitting and improve generalization ability. Table 2 details the structures and parameter settings of the embedding, recovery, generator, and discriminator networks.

During the model training phase, the CTGAN utilizes an overlapping sampling strategy (with a compensation coefficient of 400) to improve the diversity of generated data. The CTGAN successfully expands the sample size for each fault state from an initial 10 samples to 150 samples, achieving a balanced distribution with normal samples.

**Figure 7:**
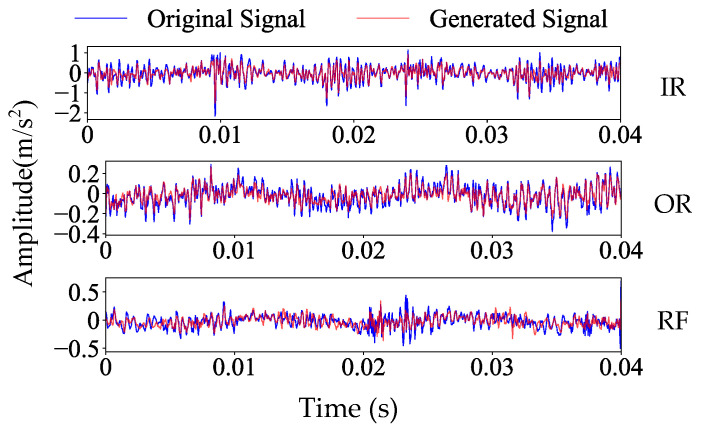
Time domain plots of the original fault signal and the generated fault signal of HUST Bearing.

To make the resemblance between the raw signals and generated signals more visually apparent, Figure 7 displays the time-domain waveforms under different fault conditions of raw signals and generated signals. Figure 7 demonstrates that compared to the raw signals, the generated signals exhibit slight differences in peak and trough amplitudes; they fully retain the glitch features of the raw signals. This ensures both class consistency and inherent variability in the generated samples.

#### 4.1.3. Ablation Experiment in the HUST Bearing Dataset

For fault diagnosis under imbalanced data, the CTGAN-MSIN method is proposed in this paper. To evaluate the contribution of each module, ablation experiments were performed on the CTGAN (a data augmentation model), MSIN (a classification module), and CTGAN-MSIN (overall model).

(a) Ablation experiments for CTGAN

Table 3 summarizes the results of the ablation test conducted on the CTGAN. According to Table 3, TimeGAN, which employs JS divergence and weight clipping, achieves a diagnostic accuracy of 92%. When JS divergence and weight clipping in TimeGAN are replaced with Wasserstein distance and gradient penalty, respectively, the accuracy of the Gradient-penalized Time-series Generative Adversarial Network (GPTGAN) increases to 95.50%, demonstrating superior optimization performance. Additionally, the Conditional Time-series Generative Adversarial Network (CTGAN), which incorporates label information into the generator and discriminator of TimeGAN, shows improved accuracy compared to TimeGAN, confirming the effectiveness of label information in diagnostic tasks. Notably, the proposed CTGAN demonstrates obvious superiority in terms of classification accuracy, and this outstanding performance fully validates the synergistic advantages of combining the condition label mechanism with the Wasserstein distance with gradient penalty strategy in enhancing fault classification performance.

(b) Ablation experiments for MSIN

The expanded dataset is fed into different classification models in the ablation experiments for performance comparison. Table 4 shows the ablation test results for MSIN. Table 4 shows that the 1D-CNN attained the poorest performance, achieving an accuracy standing at only 89.33%. After introducing the SELA module into the 1D-CNN model, the resultant CNN-ELA model exhibited a 3.93% increase in accuracy over the 1D-CNN. The MDSR model is used only for fault diagnosis with the MDSR proposed in Section 3.2. In summary, the proposed MSIN method achieves a superior diagnostic accuracy of 98.75%, outperforming all comparative approaches. Ablation test results confirm the important role of the MDSR and SELA modules in the MSIN model.

(c) Ablation experiments for CTGAN-MSIN

Table 5 presents the ablation experiment results for the CTGAN-MSIN approach. The MSIN-only model refers to fault diagnosis using MSIN directly, while the CTGAN-CNN model employs CNN for fault diagnosis based on CTGAN data augmentation. Table 5 indicates that the proposed complete method achieves the highest accuracy. Diagnostic performance significantly deteriorates when either the CTGAN data augmentation module or the MSIN module is omitted. This result further validates the effectiveness of the overall method and the complementary nature of its components.

#### 4.1.4. Diagnostic Results Analysis in the HUST Bearing Dataset

Table 2 details the specific structure and hyperparameter configuration of the CTGAN data augmentation network employed in this paper. In the classification task after data augmentation, the 150 samples for each state are split 70% for training and 30% for validation in the ensuing classification task. Additionally, the test set is composed of 100 original samples selected from each state. The hyperparameters of MSIN are listed in Table 6.

The programming code for this study is written in Python 3.9 and executed on a computer equipped with an Intel i5 CPU (manufacturer: Intel Corporation, Santa Clara, United States) and NVIDIA GeForce RTX 4070 SUPER GPU (manufacturer: NVIDIA Corporation, Santa Clara, United States).

(a) Comparison of data augmentation algorithms with other methods

**Figure 8:**
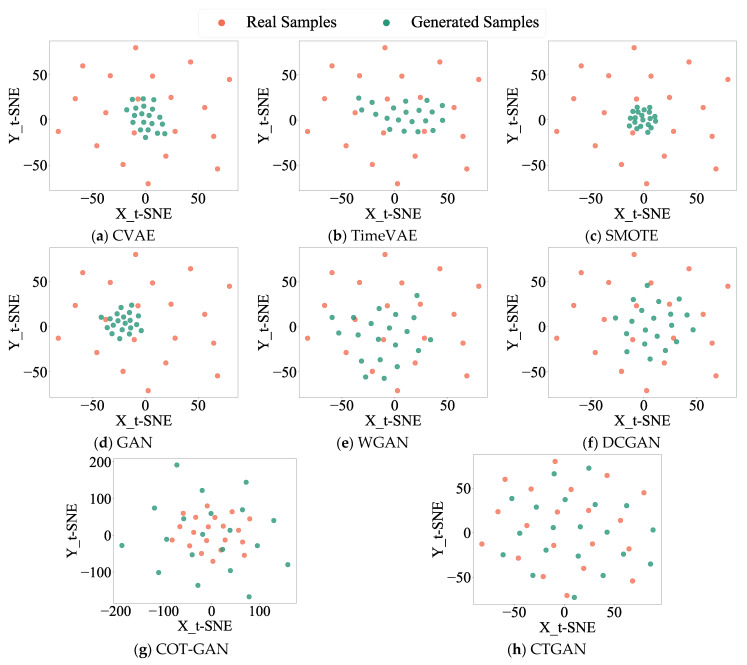
Distribution diagrams of real and generated samples in different models for HUST Bearing.

To highlight the superiority of the proposed approach, CTGAN is benchmarked against the Conditional Variational Autoencoder (CVAE) [12] and TimeVAE [38], the oversampling method SMOTE [39], and four GAN-based models: GAN [40], WGAN [18], DCGAN [16], and COT-GAN [41]. A CNN fed with the original signals is also included as a baseline. Table 7 summarizes the comparative results, presenting the average values and standard deviations obtained from ten runs. Figure 8 shows the distribution plots of real and generated data for the inner ring (IR) fault across different models.

As shown in Table 7, CTGAN achieves the best diagnostic performance with the smallest standard deviation. Apart from the baseline CNN method, CVAE yields the least favorable results. Additionally, Table 7 lists the training time for each method in one epoch. Since CNN does not involve data augmentation and SMOTE does not require designing training epochs, their training times were not calculated. From the perspective of training time, VAE-based methods (including CVAE and TimeVAE) have the shortest running time, but their accuracy and model stability are relatively poor. Among GAN-based methods, the benchmark GAN method also has the shortest running time, yet its accuracy is the lowest. Considering the three indicators of training time, accuracy, and standard deviation comprehensively, the method proposed in this paper performs more balanced and achieves relatively ideal results.

Figure 8 displays a notable divergence of the generated data distribution from the original, indicating lower-quality generated samples. The oversampling method SMOTE attains a diagnostic accuracy of 92.45% under imbalanced data conditions; however, Figure 8 shows that the generated samples of SMOTE are concentrated in a specific region, reflecting limited diversity in the generated data. Among the various GAN models, DCGAN performs the best, yet its data distribution plot still exhibits limited diversity in generated samples. Comparative analysis in Figure 8 demonstrates that the data generated by the proposed CTGAN method most closely aligns with the real data in both diversity and distribution, confirming its superior sample generation quality.

**Figure 9:**
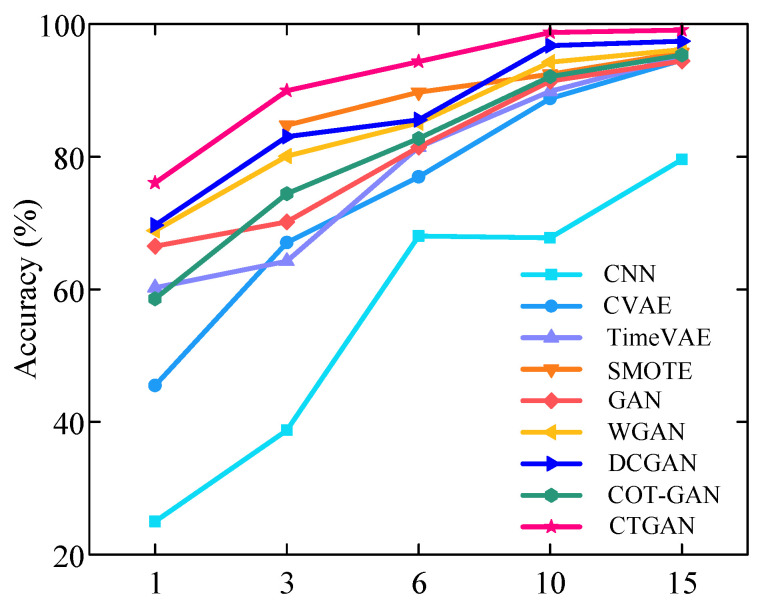
Diagnostic results under different numbers of fault samples for HUST Bearing.

Figure 9 presents the diagnostic performance results under varying numbers of fault samples. In the experiments, the size of training samples for fault state was configured as 1, 3, 6, 10, and 15, respectively, corresponding to imbalance ratios of 150:1, 50:1, 25:1, 15:1, and 10:1, respectively. As shown in Figure 9, the diagnostic accuracy of all methods shows progressive improvement with an increasing number of faults training samples, consistently outperforming the baseline CNN by a considerable margin. The proposed CTGAN achieves the best performance across all imbalance ratios. Notably, when the data imbalance ratios are 25:1, 15:1, and 10:1, the diagnostic accuracy of CTGAN exceeds 90%. In contrast, the accuracy of the comparative methods SMOTE, GAN, WGAN, DCGAN, and COT-GAN only exceeds 90% when the imbalance ratio is reduced to 15:1.

(b) Comparison of classification models with different methods

The effectiveness of the classification model is evaluated by comparing MSIN against multiple advanced methods, under two scenarios: with CTGAN-based data augmentation and without CTGAN-based data augmentation. The comparative methods include ResNet-18 [42], CNN-LSTM [43], and DPCNN [44], while a standard CNN is used as the benchmark model to maintain experimental objectivity.

**Figure 10:**
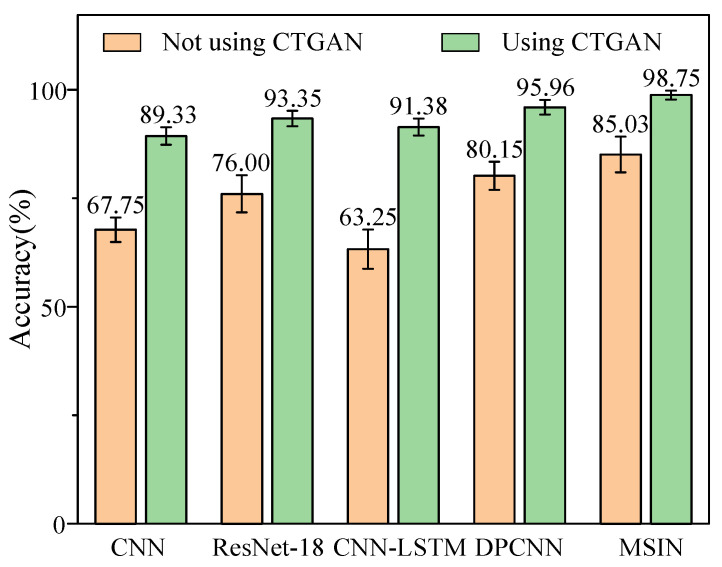
Diagnostic results of different classification models for HUST bearing.

Figure 10 presents the diagnostic results of different classification models. As can be seen from Figure 10, the MSIN model attains optimal classification results among all compared methods. Even without CTGAN-based data augmentation, MSIN attains an accuracy of 85.03%, which still significantly outperforms other methods. This result demonstrates that MSIN excels in handling fault diagnosis under imbalanced data conditions compared to other approaches. After applying CTGAN, the accuracy of all methods improves significantly. The combination of the highest accuracy along with the lowest standard deviation highlights the overall advantages of our proposed classification model of MSIN.

**Figure 11:**
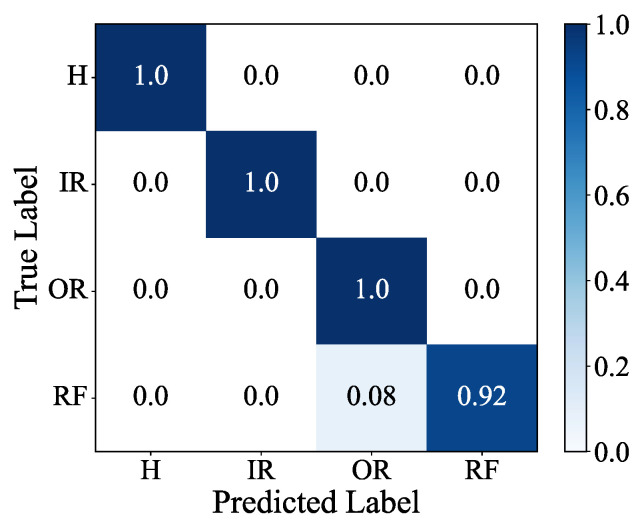
Confusion matrix in HUST bearing.

**Figure 12:**
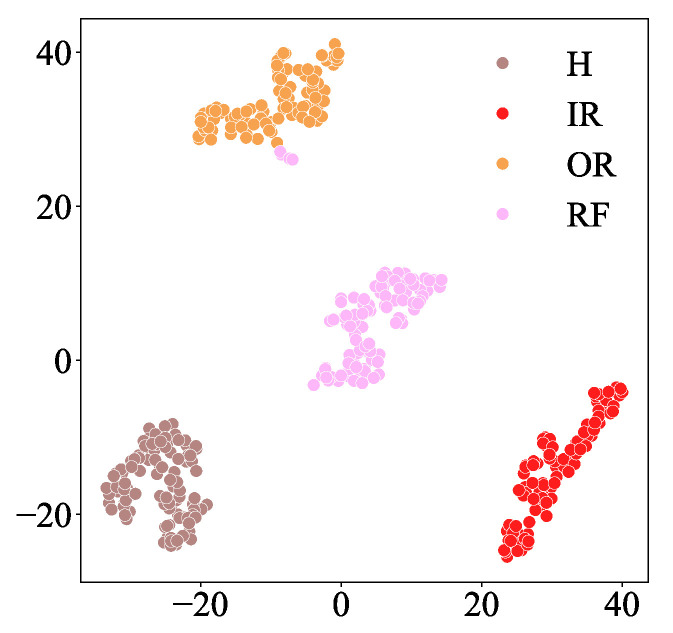
t-SNE Visualization in HUST bearing.

The confusion matrix and feature visualization results of the MSIN are provided in Figure 11 and Figure 12. The feature visualization depicted in Figure 12 demonstrates that MSIN effectively avoids mode mixing, indicating its strong robustness in feature extraction.

### 4.2. Case 2: Axial Piston Pump Dataset

#### 4.2.1. Description of Axial Piston Pump Test

**Figure 13:**
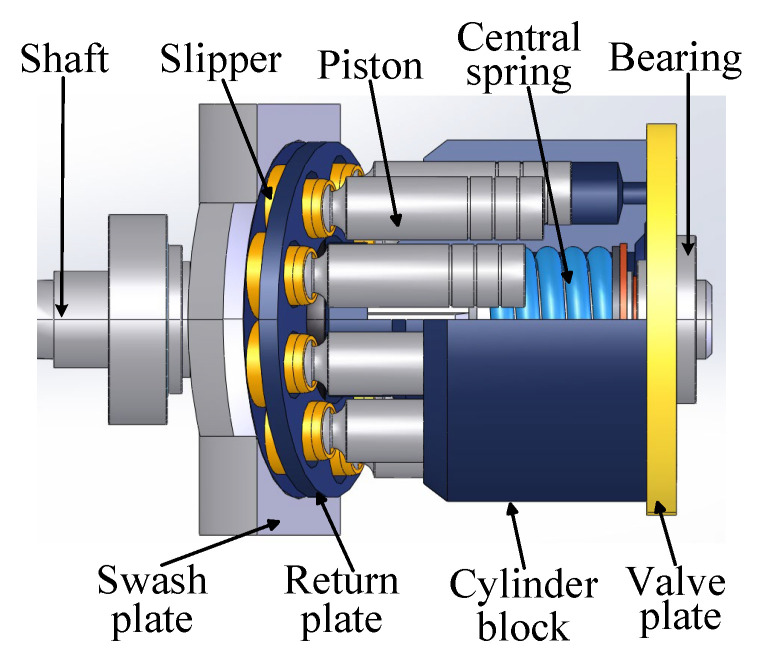
Schematic diagram of the axial piston pump.

A structural schematic diagram of the axial piston pump is depicted in Figure 13, showing components such as the shaft, swash plate, slipper, piston, cylinder block, etc. The friction pairs are essential to the axial piston pump, as they decisively influence the pump’s ability to operate properly. The main friction pairs include: (1) piston and cylinder block hole, (2) slipper and swash plate, (3) valve plate and the cylinder block’s valve plate surface, (4) slipper’s spherical socket and piston head. During long-term operation, these friction pairs inevitably experience varying degrees of wear. Therefore, common piston pump failures include piston wear, slipper wear, valve plate wear, loose slipper, and so on.

**Figure 14:**
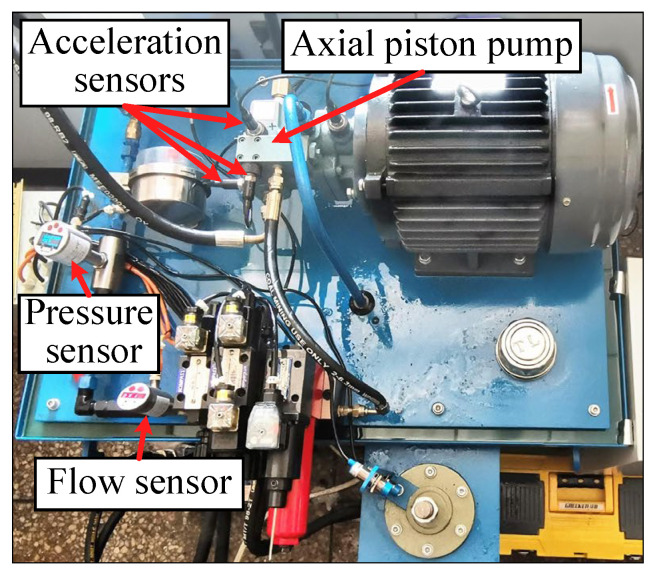
Fault diagnosis test rig of axial piston pump.

**Figure 15:**
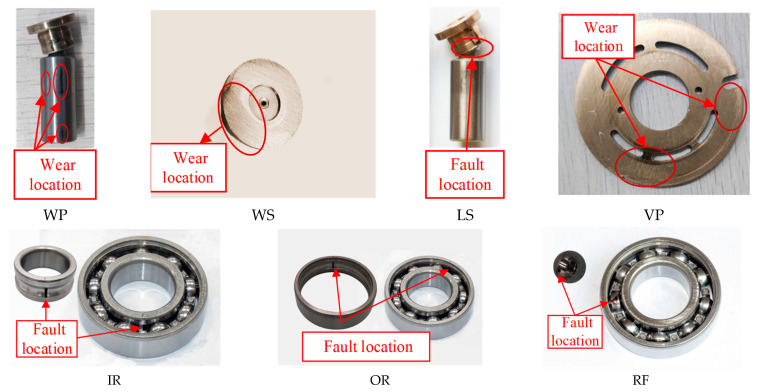
Faults in the axial piston pump.

Figure 14 shows the test bench for the axial piston pump of our laboratory. The experiments employed an axial piston pump (Model: P08-B3-F-R-01) with the following key specifications: 7 pistons, maximum displacement of 8 cm^3^/rev, and maximum pressure of 21 MPa. Based on the aforementioned study on the working mechanism of the piston pump, seven types of faults have been identified, with specific fault information listed in Table 8. Three acceleration sensors, one pressure sensor, and one flow sensor were installed during the test to monitor the operating condition of the pump. The experiment was conducted at a working pressure of 15 MPa while multi-source signals were synchronously recorded at 30 kHz. Fault diagnosis under imbalance conditions uses three vibration signals from the piston pump. To simulate data imbalance scenarios, we set the number of samples for the normal state at 150, while each fault state was assigned only 10 samples. Different faulty components of the piston pump are identified in Figure 15.

#### 4.2.2. Data Generation and Evaluation in the Axial Piston Pump Dataset

**Figure 16:**
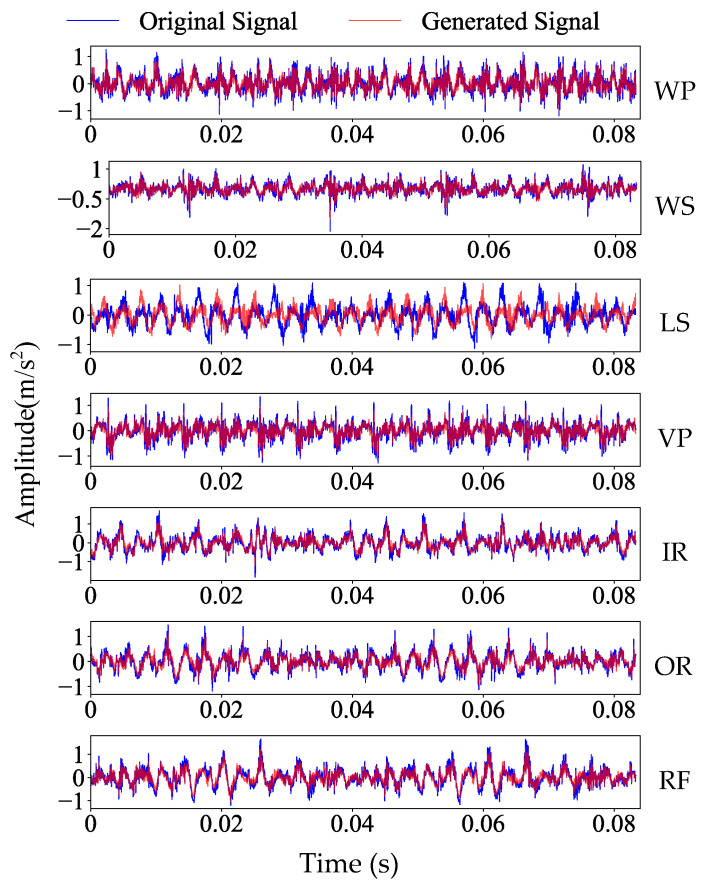
Time domain diagram of raw fault signals and generated fault signals of axial piston pump.

In this section, CTGAN is used to perform data augmentation for each fault state, and the network architecture and hyperparameter configuration are shown in Table 2. Figure 16 presents a visualization comparing the raw fault signal data with generated data from the axial piston pump. Figure 16 shows that the generated signals are highly similar to the raw signals, although they are not identical.

#### 4.2.3. Ablation Experiment in the Axial Piston Pump Dataset

This section conducts confusion experiments on the axial piston pump dataset to evaluate the contribution of each module to fault diagnosis. Table 9, Table 10 and Table 11 present the ablation test results for CTGAN, MSIN, and the model CTGAN-MSIN on this dataset, respectively. The ablation test in Table 9 and Table 10 demonstrates that both the CTGAN and MSIN modules designed in this paper exhibit significant effectiveness. From Table 11, it can be observed that the diagnostic accuracy shows a significant decline in the CTGAN-MSIN model when either the CTGAN or the MSIN module is absent.

#### 4.2.4. Diagnostic Results Analysis in the Axial Piston Pump Dataset

(a) Comparison of data augmentation algorithms with other methods

**Figure 17:**
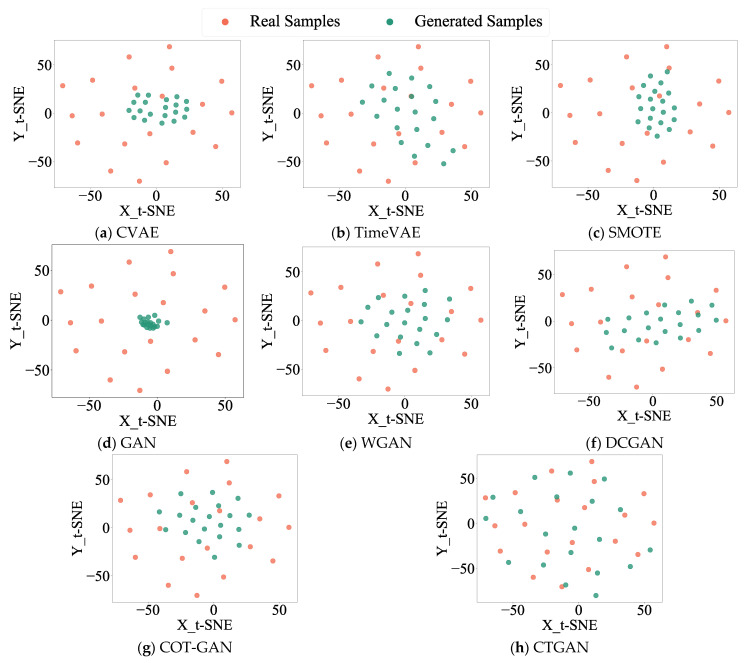
Distribution diagrams of real and generated samples in different models for the axial piston pump.

The CTGAN is compared with other advanced methods using experimental data from an axial piston pump. The architecture and hyperparameters of CTGAN are shown in Table 2, and the hyperparameter configuration of MSIN is shown in Table 6. Table 12 presents the comparative results. Taking the IR fault as an example, Figure 17 displays the distribution plots of real versus generated fault data from different models. Consistent with the conclusions in Section 4.1.4(a), the proposed CTGAN demonstrates superior diagnostic performance with the smallest standard deviation under data-imbalanced scenarios. Among all compared methods, the traditional GAN performs the least satisfactorily, except for the baseline CNN method. The GAN’s distribution plot in Figure 17 reveals clustered generated samples, indicating limited diversity in generated data. DCGAN achieves the best performance among conventional methods, yet its accuracy reaches only 91.75% with a relatively larger standard deviation compared to CTGAN. As shown in Figure 17, the generated data of CTGAN maintains a consistent distribution with raw data, while other methods exhibit issues of generated sample clustering and distribution inconsistency.

**Figure 18:**
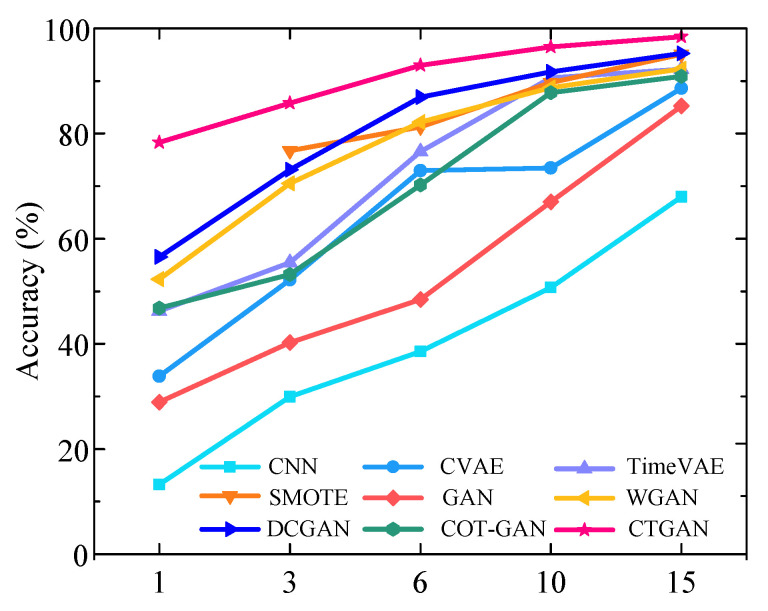
Diagnostic results under different numbers of fault samples for axial piston pump.

Diagnostic results of the piston pump under varying numbers of fault samples are presented in Figure 18. As shown in Figure 18, the accuracy of all methods improves with a decrease in the class imbalance ratio. The proposed CTGAN method achieves optimal diagnostic performance under imbalanced data conditions, maintaining accuracy above 90% when the imbalance ratio is below 25:1.

(b) Comparison of classification models with different methods

**Figure 19:**
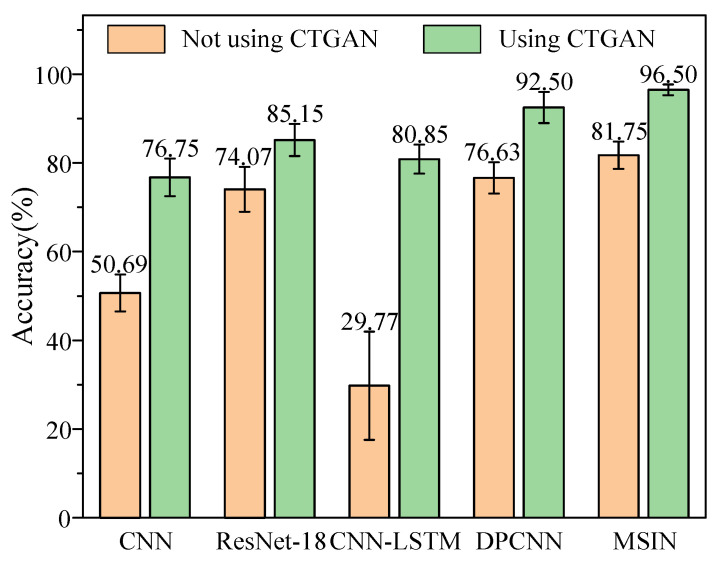
Diagnostic results of different classification models for axial piston pump.

**Figure 20:**
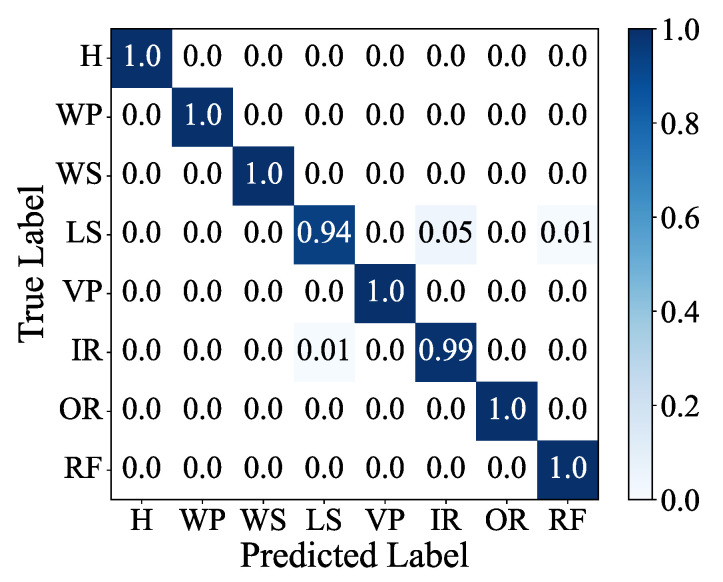
Confusion matrix in axial piston pump.

**Figure 21:**
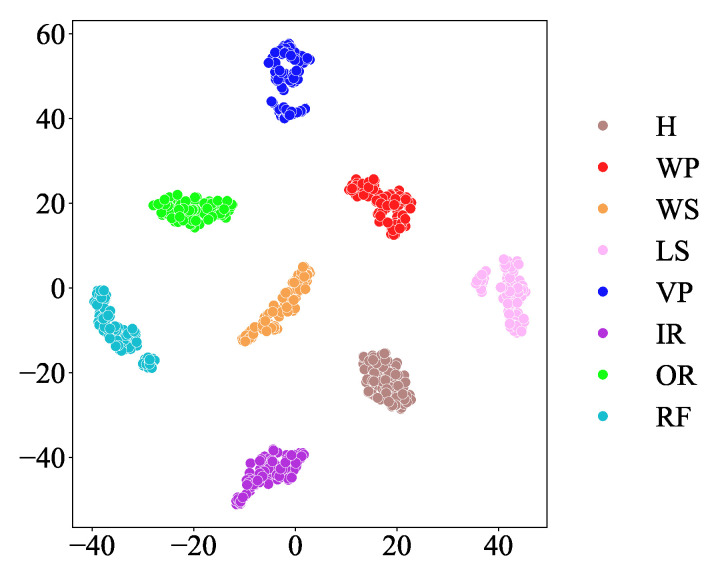
t-SNE Visualization in axial piston pump.

Figure 19 presents a comparative analysis of different classification models in axial piston pump fault diagnosis, revealing the following key findings: First, when employing CTGAN for data augmentation, all classification models demonstrated significantly better diagnostic performance compared to scenarios without data augmentation. Second, the proposed MSIN model consistently achieved optimal performance regardless of whether CTGAN data augmentation was applied, attaining not only the highest diagnostic accuracy but also maintaining the smallest standard deviation. These experimental results comprehensively verify MSIN’s superior performance and strong robustness in fault diagnosis tasks. Figure 20 and Figure 21 present the confusion matrix and feature visualization diagrams, respectively, of the proposed CTGAN-MSIN method.

## 5. Conclusions

This paper proposes a CTGAN-MSIN to address fault diagnosis under imbalanced data in rotating machinery. CTGAN is employed to expand the imbalanced data, while MSIN is used for fault diagnosis after data augmentation. On the one hand, the CTGAN introduces the Wasserstein distance and gradient penalty as loss functions to construct a more stable adversarial training framework. Meanwhile, CTGAN achieves controllable generation of fault type samples by adding label conditions, ultimately outputting diverse and high-quality multi-source fault data. On the other hand, MSIN extracts fault features at different scales and incorporates a SELA module to address the issue of the varying importance of features at different scales. This enables the precise selection of more discriminative scale features, thereby ensuring the robustness of the diagnostic performance. Tests of the HUST bearing and the self-collected axial piston pump dataset demonstrate the superior performance of CTGAN-MSIN. Through comparative experiments on data augmentation methods and classification models, the proposed method achieved optimal accuracy, demonstrating the significant advantages of CTGAN in data augmentation and the strong capability of MSIN in fault diagnosis. Moreover, CTGAN-MSIN exhibits excellent classification performance under different data imbalance ratios. Future efforts will focus on utilizing algorithms to fine-tune the hyperparameters of the CTGAN to enhance its data generation quality.

However, CTGAN still possesses significant potential for optimization. On the one hand, the current model parameters are all manually configured; subsequent fine-tuning of its hyperparameters using optimization algorithms could further enhance the quality of generated data. On the other hand, the training time of CTGAN is relatively long, and future work could explore lightweight designs for generative adversarial networks to reduce computational costs.

## Figures and Tables

**Table 1:** HUST bearing fault dataset description.

Fault Type	Operating Condition	Label	Sample Number
Health	3900 rpm	H	150
Inner ring fault	3900 rpm	IR	10
Outer ring fault	3900 rpm	OR	10
Rolling element fault	3900 rpm	RF	10

**Table 2:** The structure and parameters of the CTGAN.

Network	Structure	Parameter	
Embedder	GRU	Hidden_dim = 24	Optimizer = Adam Initial learning rate = 0.0001 Decay steps = 100 Decay rate = 0.96 Epochs = 2000
	Dropout	Dropout rate = 0.2	
	GRU	Hidden_dim = 24	
	Dense	Units = 24	
	Layer Norm	*β* = 0, *γ* = 1	
Recovery	GRU	Hidden_dim = 24	
	Dropout	Dropout rate = 0.2	
	GRU	Hidden_dim = 24	
	Dense	Units = 24	
	Layer Norm	*β* = 0, *γ* = 1	
	Dense1	Units = 3	
Generator	GRU	Hidden_dim = 24	
	Dropout	Dropout rate = 0.2	
	Dense1	Units = 3	
Discriminator	GRU	Hidden_dim = 24	
	Dropout	Dropout rate = 0.2	
	Dense	Units = 24	
	Layer Norm	*β* = 0, *γ* = 1	
	Dense2	Units = 1	

**Table 3:** The ablation experiment results of the CTGAN in the HUST bearing dataset.

Model	TimeGAN	Condition Label	Wasserstein Distance with Gradient Penalty	Accuracy (%)
TimeGAN	√	×	×	92.00
CTGAN (without GP)	√	√	×	96.86
GPTGAN	√	×	√	95.50
CTGAN	√	√	√	98.75

**Table 4:** The ablation experiment results of the MSIN in the HUST bearing dataset.

Model	1D-CNN	MDSR	ELA	SELA	Accuracy (%)
MDSR	×	√	×	×	94.73
CNN-ELA	√	×	√	×	93.26
1D-CNN	√	×	×	×	89.33
MSIN	×	√	×	√	98.75

**Table 5:** The ablation experiment results of the CTGAN-MSIN in the HUST bearing dataset.

Model	CTGAN	MSIN	CNN	Accuracy (%)
MSIN-only	×	√	×	85.03
CTGAN-CNN	√	×	√	89.33
CTGAN-MSIN	√	√	×	98.75

**Table 6:** The hyperparameters of MSIN.

Main Parameters	Values
Batch size	16
Optimizer	Adam
Learning rate	0.0001
Training epochs	100

**Table 7:** Comparative diagnostic results of data augmentation methods for the HUST bearing.

Method	Accuracy (%)	Precision (%)	Recall (%)	F1-Score (%)	Time (s)
CNN	67.75 ± 2.82	73.56 ± 3.69	67.75 ± 2.82	65.61 ± 3.75	——
CVAE	88.75 ± 8.56	90.93 ± 6.77	88.75 ± 8.56	87.04 ± 10.99	0.0215
TimeVAE	89.86 ± 4.59	91.63 ± 2.40	89.86 ± 4.59	89.07 ± 5.83	0.0070
SMOTE	92.45 ± 3.32	92.82 ± 3.37	92.45 ± 3.32	92.35 ± 3.37	——
GAN	91.39 ± 5.35	93.14 ± 3.45	91.39 ± 5.35	90.10 ± 6.55	0.3119
WGAN	94.25 ± 3.89	95.30 ± 3.1	94.25 ± 3.89	94.19 ± 4.10	3.0125
DCGAN	96.75 ± 1.45	96.83 ± 2.12	96.75 ± 1.45	96.72 ± 2.16	0.6826
COT-GAN	92.08 ± 4.91	92.74 ± 5.45	92.08 ± 4.91	92.70 ± 3.73	5.4786
CTGAN	98.75 ± 1.19	98.66 ± 1.30	98.75 ± 1.19	98.48 ± 1.46	5.8146

**Table 8:** Types of axial piston pump failures.

Fault Type	Label	Sample Number
Health	H	150
Wear of piston	WP	10
Wear of slipper	WS	10
Loose slipper	LS	10
Wear of valve plate	VP	10
Inner ring fault	IR	10
Outer ring fault	OR	10
Rolling element fault	RF	10

**Table 9:** The ablation experiment results of the CTGAN in the axial piston pump dataset.

Model	TimeGAN	Condition Label	Wasserstein Distance with Gradient Penalty	Accuracy (%)
TimeGAN	√	×	×	90.86
CTGAN (without GP)	√	√	×	94.50
GPTGAN	√	×	√	93.25
CTGAN	√	√	√	96.50

**Table 10:** The ablation experiment results of the MSIN in the axial piston pump dataset.

Model	1D-CNN	MDSR	ELA	SELA	Accuracy (%)
MDSR	×	√	×	×	91.50
CNN-ELA	√	×	√	√	88.68
1D-CNN	√	×	×	×	76.75
MSIN	×	√	×	√	96.50

**Table 11:** The ablation experiment results of the CTGAN-MSIN in the axial piston pump dataset.

Model	CTGAN	MSIN	CNN	Accuracy (%)
MSIN-only	×	√	×	81.75
CTGAN-CNN	√	×	√	76.75
CTGAN-MSIN	√	√	×	96.50

**Table 12:** Comparative diagnostic results of data augmentation methods for axial piston pump.

Method	Accuracy (%)	Precision (%)	Recall (%)	F1-Score (%)	Time (s)
CNN	50.69 ± 4.16	61.23 ± 2.78	50.69 ± 4.16	48.06 ± 4.12	——
CVAE	73.47 ± 5.83	75.94 ± 7.99	73.47 ± 5.83	70.93 ± 5.61	0.0378
TimeVAE	90.63 ± 4.78	91.02 ± 5.48	90.63 ± 4.78	90.76 ± 4.20	0.0191
SMOTE	89.69 ± 5.46	91.44 ± 5.08	89.69 ± 5.46	89.42 ± 5.72	——
GAN	67.00 ± 7.75	61.90 ± 9.42	67.00 ± 7.75	61.37 ± 10.23	0.3777
WGAN	88.75 ± 6.10	92.59 ± 5.78	88.75 ± 6.10	85.78 ± 6.12	3.5187
DCGAN	91.75 ± 4.83	93.19 ± 2.12	91.75 ± 4.83	91.33 ± 5.12	0.8125
COT-GAN	87.75 ± 3.53	88.40 ± 4.60	87.75 ± 3.53	88.37 ± 2.80	6.0504
CTGAN	96.50 ± 1.22	96.84 ± 0.82	96.50 ± 1.22	96.37 ± 1.59	6.3361

## Data Availability

Data is unavailable due to privacy.
